# Learning rich features with hybrid loss for brain tumor segmentation

**DOI:** 10.1186/s12911-021-01431-y

**Published:** 2021-07-30

**Authors:** Daobin Huang, Minghui Wang, Ling Zhang, Haichun Li, Minquan Ye, Ao Li

**Affiliations:** 1 School of Information Science and Technology, and Centers for Biomedical Engineering, University of Science and Technology of China, Hefei, 230027 China; 2 School of Medical Information, Wannan Medical College, Wuhu, 241002 China; 3 Research Center of Health Big Data Mining and Applications, Wannan Medical College, Wuhu, 241002 China; 4 Department of Biochemistry, Wannan Medical College, Wuhu, 241002 China

**Keywords:** Brain tumor segmentation, Rich feature representation, Deep learning, Class imbalance

## Abstract

**Background:**

Accurately segment the tumor region of MRI images is important for brain tumor diagnosis and radiotherapy planning. At present, manual segmentation is wildly adopted in clinical and there is a strong need for an automatic and objective system to alleviate the workload of radiologists.

**Methods:**

We propose a parallel multi-scale feature fusing architecture to generate rich feature representation for accurate brain tumor segmentation. It comprises two parts: (1) Feature Extraction Network (FEN) for brain tumor feature extraction at different levels and (2) Multi-scale Feature Fusing Network (MSFFN) for merge all different scale features in a parallel manner. In addition, we use two hybrid loss functions to optimize the proposed network for the class imbalance issue.

**Results:**

We validate our method on BRATS 2015, with 0.86, 0.73 and 0.61 in Dice for the three tumor regions (complete, core and enhancing), and the model parameter size is only 6.3 MB. Without any post-processing operations, our method still outperforms published state-of-the-arts methods on the segmentation results of complete tumor regions and obtains competitive performance in another two regions.

**Conclusions:**

The proposed parallel structure can effectively fuse multi-level features to generate rich feature representation for high-resolution results. Moreover, the hybrid loss functions can alleviate the class imbalance issue and guide the training process. The proposed method can be used in other medical segmentation tasks.

## Background

Accurately segment the tumor region of MRI images is a key step in radiation therapy for brain cancer [1]. Brain tumors are the result of uncontrolled proliferation of cancer cells in the brain. In general, tumor shapes and locations in the brain are different from patient to patient [2], making it hard to annotate tumor areas for clinical purposes or radiotherapy planning. At present, manual segmentation is wildly adopted in clinical, but its accuracy and reliability depend on the slice reading ability of radiologists. Therefore, there is a strong need for an automatic and objective system to alleviate such demanding.

In recent decades, researchers have proposed many automatic methods to segment brain tumors, including discriminative and generative approaches [1]. The generative approaches depend on the specific domain knowledge, such as the appearance characteristics of tumor areas and its surrounding areas. In general, prior knowledge of the target tumor region is difficult to code. Compared with the image signal or expected shape of normal tissues, the existing generative methods model tumors as outliers [3–5]. Tumors may appear with different sizes and complex shapes, so aligning a brain with tumors onto a template can be challenging. To get a better segmentation performance, researchers have proposed different methods for segmenting brain images with tumors and registering these images to a template computed from normal brains [6–8]. Unlike generative approaches, the discriminative approaches use little prior domain knowledge and their implementation depends on the engineered or hand-crafted features.

At present, convolutional neural network is the most popular discriminative approach for multimodal brain image segmentation [9–14]. For example, Havaei et al. [13] present a patch-wise CNN architecture with a two-pathway structure to segment brain tumors, which use local and global contextual features. Zhao and Jia [14] design a patch-based model with three-pathway streams. They show higher accuracy and robustness compared with traditional CNNs. Zhao et al. [11] propose a patch-based model and achieve the best results in BRATS 2015. However, the main issue of patch-based methods is that the training process of the model are inefficient [15] and does not take into account the effect of the whole image [16]. Currently, U-net is widely applied for medical image segmentation, and researchers propose many networks extending the architecture, such as 3D U-net [17] and V-net [18]. Although 3D input data provide more semantic information, there are large parameters in 3D CNN, so more memory and computing resources are needed.

Despite the great progress achieved by the aforementioned studies, automated brain segmentation remains a challenging task for the following factors. First, brain tumors vary in size and shape, so a rich feature representation with a high-resolution level is needed for the precise segmentation of tumor sub-regions. Specifically, effective feature extraction and feature fusion is the key to achieving good performance. Second, the severe class imbalance is another factor that will harm the training process and impact on performing segmentation. When the methods based on deep learning optimize with cross-entropy loss or Dice loss, non-tumor regions will dominate the optimization process of the neural network.

We do not apply complex pre-processing or any post-processing steps, but focus on designing a simple network structure which can perform effective feature extraction and feature fusion for rich feature representation. Inspired by the design philosophy of Hypercolumn [19], we extend the pixel-wise prediction task and design a simple parallel convolutional neural network, which can split the whole brain into different sub-regions. For the severe class imbalance issue, we design two hybrid loss functions, which include recall loss, combined Dice loss, and cross-entropy loss. Our method test on BRATS 2015 and achieve promising results in labeling different sub-regions of the tumor.

In this paper, the contributions are three aspects:

1. We propose a simple parallel Convolutional Architecture (the size of parameters is only 6.3 MB) that extracts different-level features and parallel fusing those features for rich feature representation.
2. We design two hybrid loss functions to alleviate the severe class imbalance issue, which can effectively guide the training process.
3. Without any post-processing operations, our method still outperforms published state-of-the-arts methods on the segmentation results of complete tumor regions and obtains competitive performance in another two regions.

## Methods

### Datasets and pre-processing

There are two data sets in BRATS 2015, one for testing and the other for training. For the training dataset, it comprises 274 cases (low grade tumors: 54, high grade tumors: 220), all of which are annotated at the pixel level. For the testing dataset, it contains 110 cases without ground truth (the number of low and high grade tumors is not disclosed). Each case comprised 4 MRI sequences: T2, T1-contrast, T1, and FLAIR. The dimensions of each sequence are 240 × 240 × 155. All sequences of the same case had been co-registered. The manual segmentations of each case are labeled with 5 different numbers: enhancing tumor: 4, non-enhancing tumor: 3, edema: 2, necrosis: 1, and 0 for everything else.

**Fig. 1:**
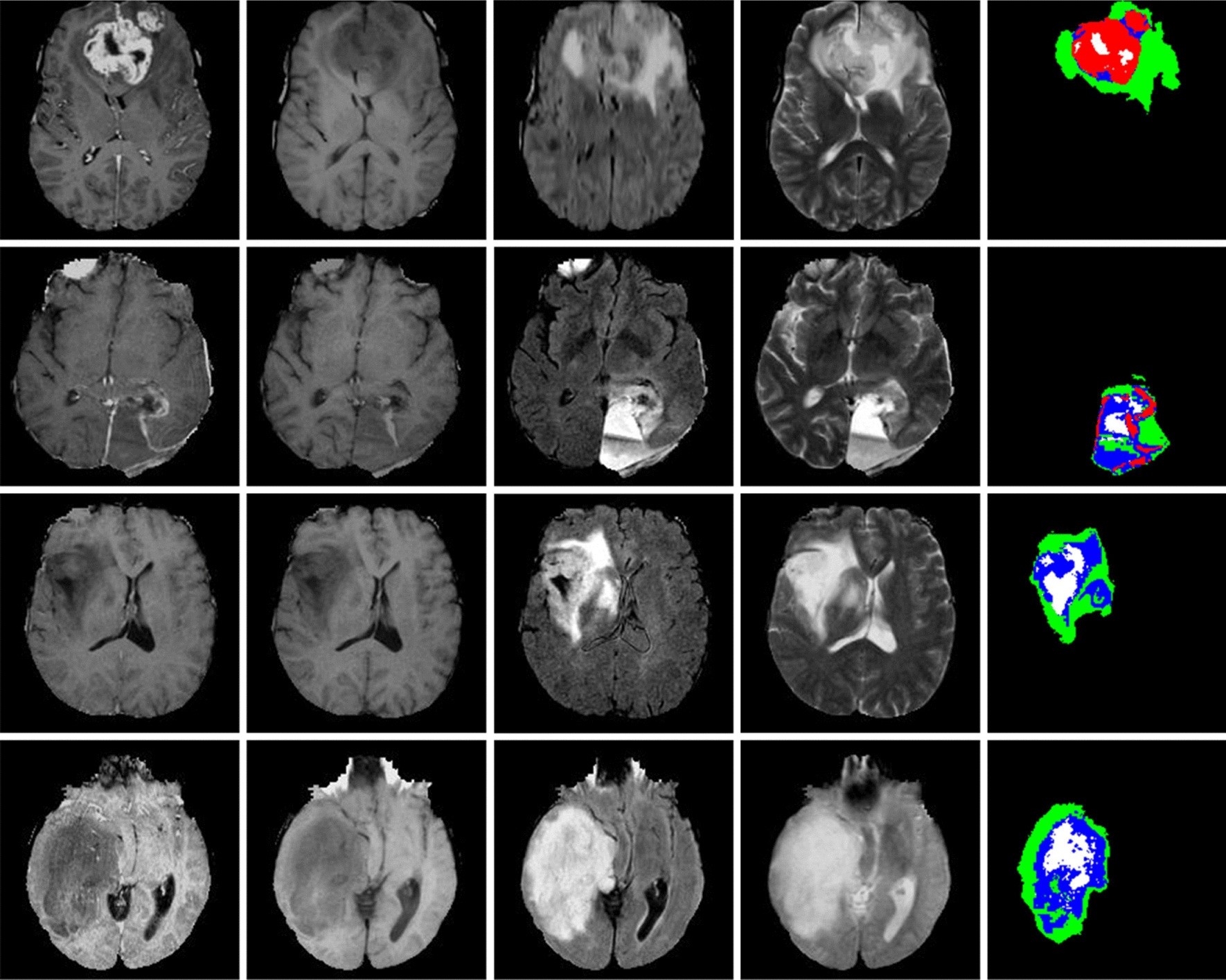
Examples of the BRATS 2015 training set (best view in color), showing two HGG cases in the first two rows and two LGG cases in the last two rows. Each row represents (from left to right): T1c, T1, FLAIR, T2 and ground truth. Pixels labeled in black are background in the last column. Each of the other colors represents a tumor region: necrosis (blue), edema (green), non-enhancing (white) and enhancing (red)

Figure 1 shows four slices from different cases and its corresponding ground truth. We evaluate all the predictions results for the test set via an online system. It requires evaluating three different tumor regions: complete tumor (labels 4 + 3 + 2 + 1), tumor core (labels 4 + 3 + 1) and enhancing tumor (label 4). We crop each case into a volume of 176 × 176 × 155, removing the border area while keeping the entire area of the brain. After that, we normalize the brain MRI images to have unit variance and zero-mean.

### The proposed network architecture

**Fig. 2:**
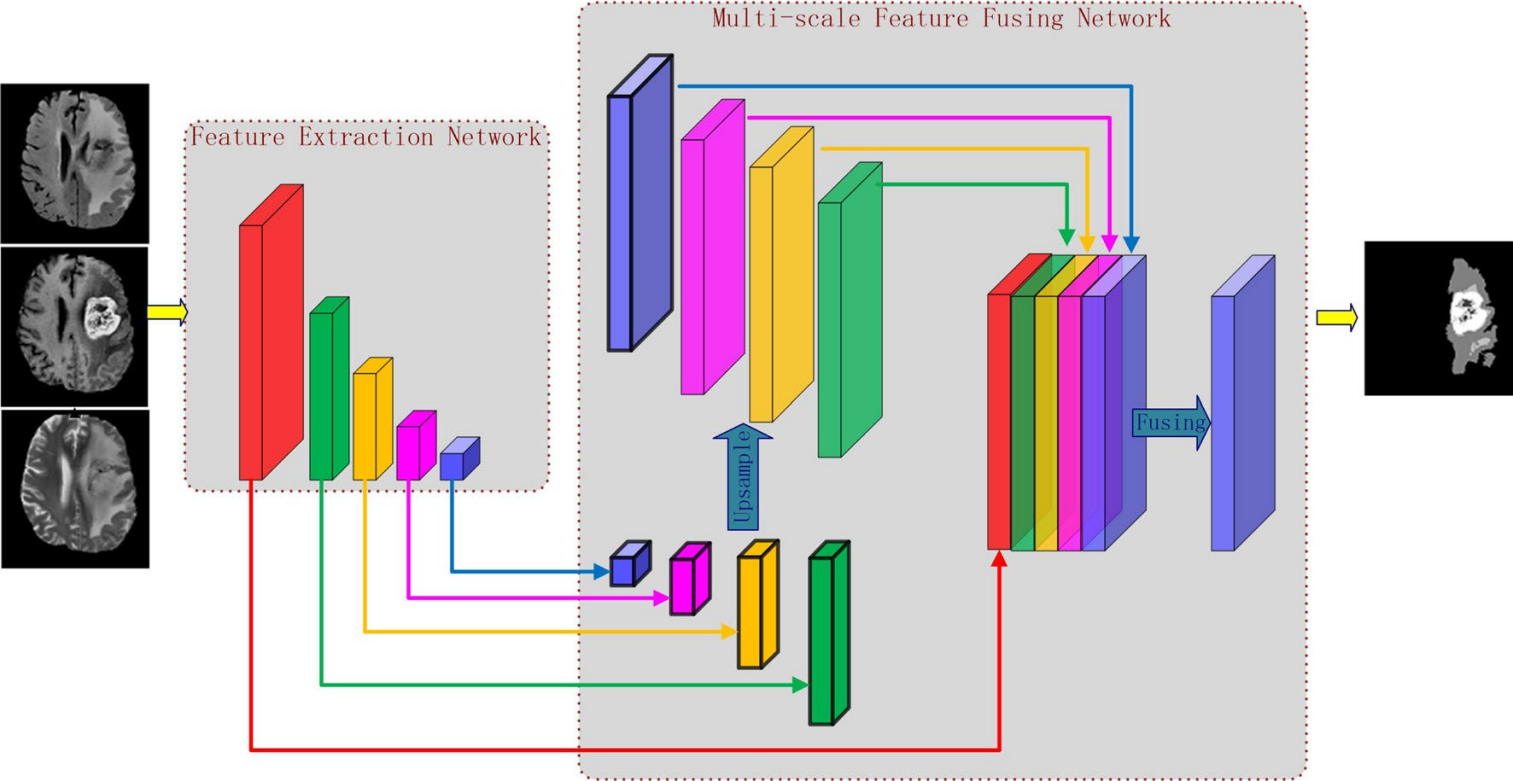
The architecture of the proposed model (best view in color). Each block in Feature Extraction Network represents feature maps with different size (from left to right): 256*256, 128*128, 64*64, 32*32 and 16*16. In the multi-scale feature fusion network, the multi-scale feature maps are first up-sampled to the same size (176 × 176), and then fused

We propose a parallel CNN architecture that differs from U-net used in medical image. As shown in Fig. 2, the proposed architecture comprises two parts: the Feature Extraction Network (FEN) and the Multi-scale Feature Fusing Network (MSFFN). The feature extract part comprises twelve convolution layers for getting multi-level features and four max-pooling layers for enlarging the receptive field of the whole network. In the feature fusing part, multi-level feature generated by the extraction part is up-scaled to the original size (176 × 176), and then parallel fed into the feature fusing block. We introduce more details about the two networks in the following subsections.

#### Feature extraction network

The multi-scale feature representation is of crucial importance for the medical segmentation task, especially when the target region with different sizes. In general, the performances are not superior when tumor tissues with small size or complex topology. In a convolutional neural network, the feature maps get from deeper convolution layers contain more semantic information, but the feature maps with high-resolution contain more spatial details. Moreover, the detail information for small segmentation targets will be lost in the deeper layers and can only obtain the fine details in the early layers.

**Fig. 3:**
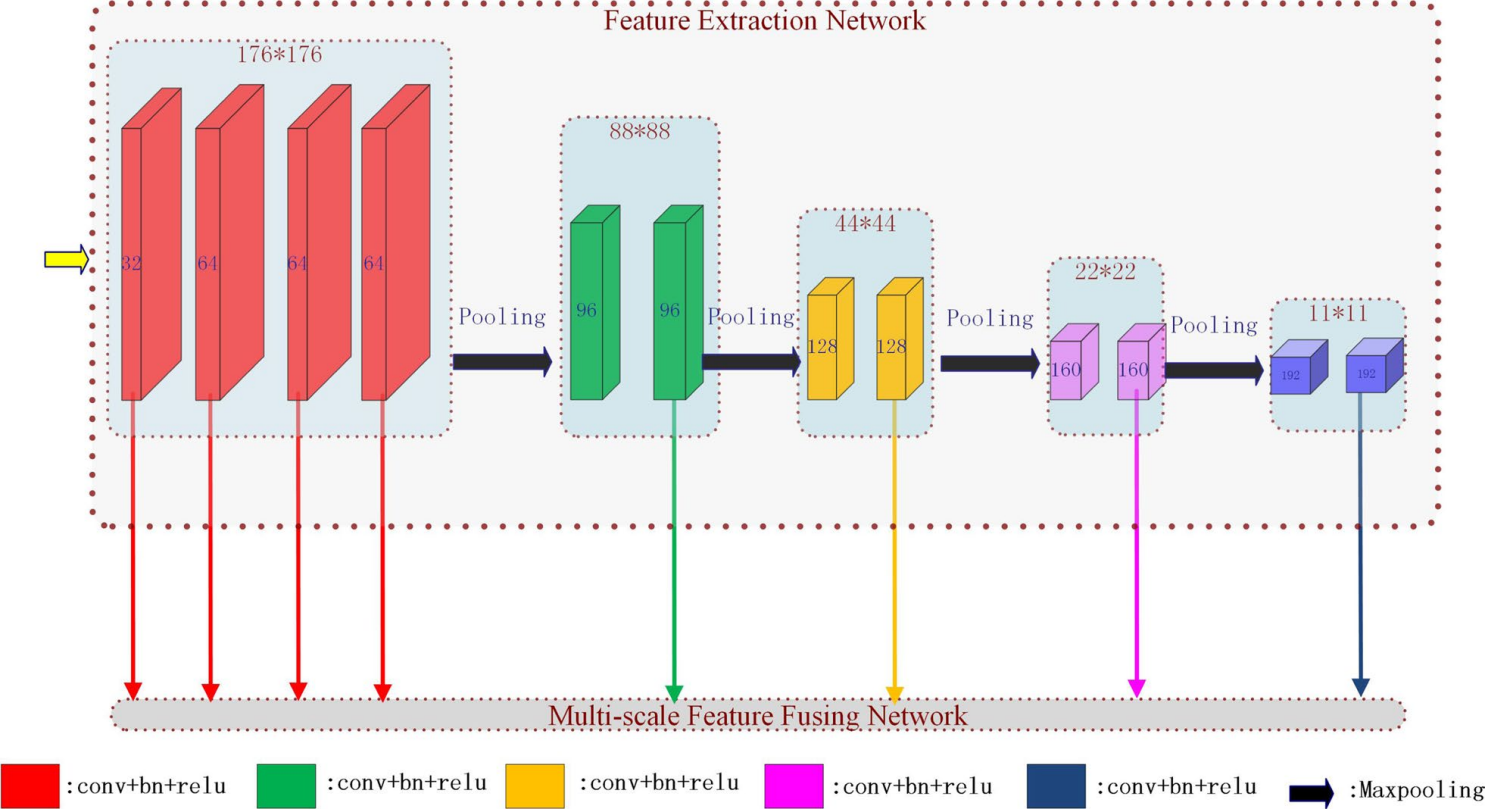
The architecture of feature extraction network (best view in color)

As shown in Fig. 3, FEN is divided into five stages. Each stage in the network uses two convolution units to extract multi-level semantic information for MSFFN, except the first stage, which uses four convolution units. Each unit includes a convolution layer (stride: 1, kernel: 3 × 3), a BN layer, and an activation layer (ReLU). At the end of each stage (except for the last one), there are a max-pooling layer (stride: 2, kernel size: 2 × 2) and a dropout layer.

#### Multi-scale feature fusing network

There are many ways to fuse multi-scale features for semantic segmentation. The first way is merging multiple top features with different scales output by the backbone network and then forming new feature maps with more information, such as DeepLab [20] and PSPNet [21]. The other way is gradually fusing multi-level features from top layers to bottom layers, such as U-net [22], SegNet [23] and RefineNet [24]. This feature fusion mechanism is widely used, especially in medical images, but its working mechanism is not clear. Furthermore, Zhang [25] point out that current U-net architectures have an ineffective feature fusion problem and proposed a framework named ExFuse [25].

As seen in Fig. 3, all feature maps extracted from different convolution units in FEN can be fed into MSFFN. Our feature fusing network is inspired by the philosophy of Hypercolumn descriptors [19]. In general, the coarse high-level features representation contains more semantic information, while low-level ones carry more detail information important to segment small objects. To reduce computation time and memory cost, we only chose the feature maps generated by a convolution layer at the end of each stage in FEN except for the first stage. The reason we take all the feature maps in the first stage is that those low-level but high-resolution features have a more powerful representation in detail information.

**Fig. 4:**
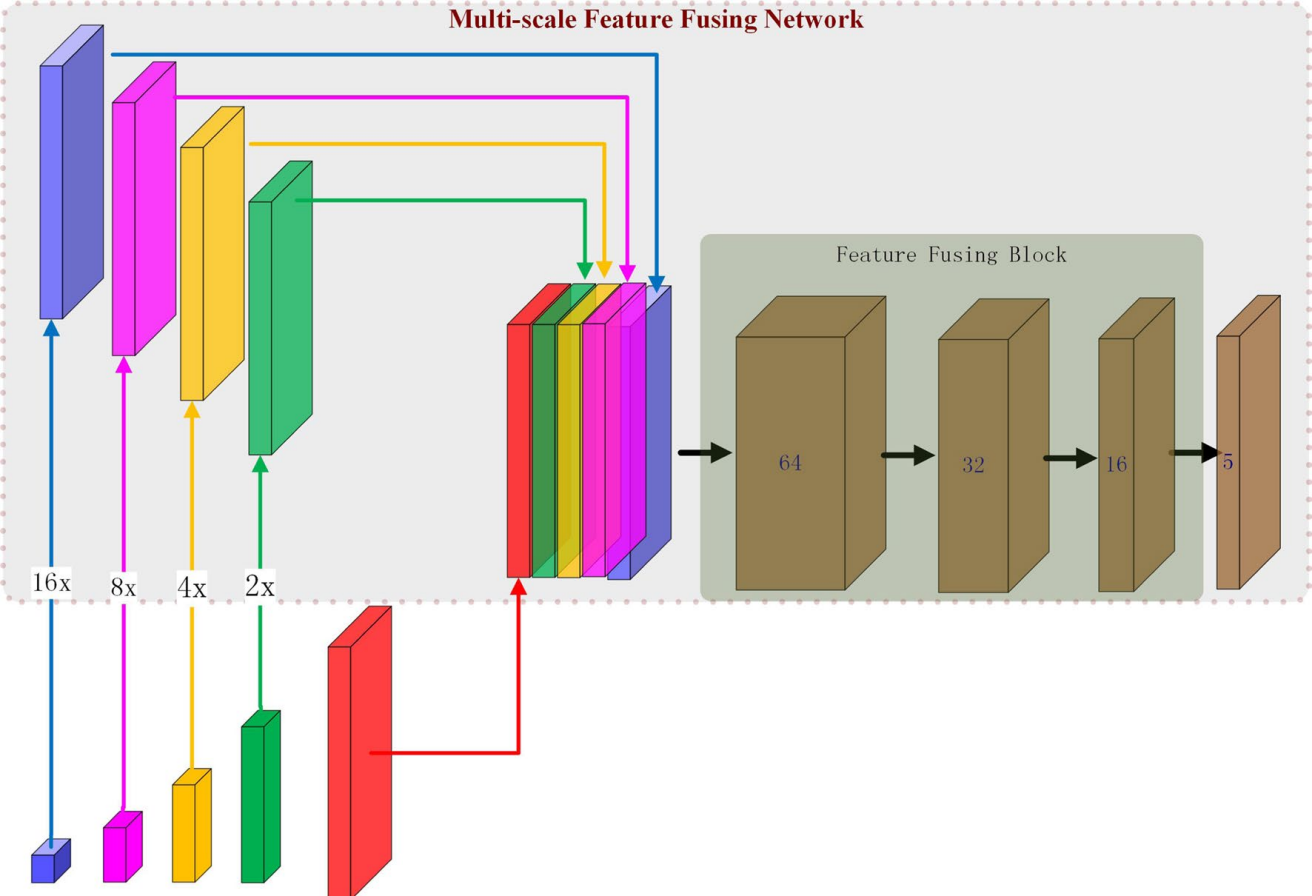
The architecture of Multi-scale Feature Fusing Network (best view in color). 16×, 8×, 4×, and 2× represent the up sampling factors for feature maps of sizes 16 * 16, 32 * 32, 64 * 64, and 128 * 128, respectively. The Feature Fusing Block includes three convolution units, each composed of a convolution layer, a BN layer and an activation layer

Figure 4 shows an overview of our proposed feature fusing network. It employs a parallel branching structure to concatenate multi-scale feature maps. Since MSFFN takes feature maps with different scales in FEN as input, we apply the up-sampling operation to the low- resolution semantic feature maps to generate feature maps with the size like original images (176 × 176) via bilinear interpolation. The reason we use bilinear interpolation is that it can reduce the model parameters and facilitate the gradient propagation to the deep convolutional layer, to realize the effective training of the whole network. The next step is to concatenate features of the equivalent size together. The concatenated features then go through the feature fusing block that aims to efficiently integrate multi-level features, which is expected to narrow the gap between the different features and make full use of those features. In particular, this component includes three convolution units, each composed of a convolution layer (stride: 1, kernel: 3 × 3), a BN layer and an activation layer (ReLU). There is a dropout layer after the feature fusing block. Finally, a Softmax layer receives feature maps generated by the feature fusion block to make the prediction.

### Loss function and class imbalance

Class imbalance is very common and has attracted much attention in recent years [26]. For BRATS 2015, the pixels of different regions are extremely unbalanced. The network training on such datasets, with low recall and high accuracy prediction, is biased towards background areas in medical applications [27, 28, 30, 31, 33–35]. Therefore, some researchers propose algorithm-level approaches to solve this issue [17, 18, 22, 27–34], such as weighted cross-entropy loss function. Dice loss [18], generalized Dice loss [31], asymmetric similarity loss [28], hybrid loss with cross-entropy and Dice loss [33] and combo loss [30]. For the class imbalance, we propose two hybrid loss functions comprising contributions from different losses, including recall loss, combined Dice loss and cross-entropy loss. Let G be ground truth (with size 176 × 176 × 5), P be the predicted results (with size 176 × 176 × 5).

#### Cross-entropy loss

We can write the cross-entropy loss for a multi-class task as:

1 $${L}_{ce\_loss}=-\frac{1}{N}\sum_{i=1}^{N} \sum_{k=0}^{K-1}{g}_{i,k}log{p}_{i,k}$$

where ${\mathrm{g}}_{\mathrm{i},\mathrm{k}}\in \left\{\mathrm{0,1}\right\}$ and ${\mathrm{p}}_{\mathrm{i},\mathrm{k}}$∈[01] denote the manual annotation and the predictions of the Softmax at each pixel i for class k, N represents the number of pixels.

#### Dice loss

There are many criteria for evaluating image segmentation algorithms, among which Dice similarity coefficient (DSC) is the most widely applied. Milletari et al. [18] propose a differentiable version of it to optimize the model. The Dice loss for each subclass can be expressed as:

2 $${\text{D}}L_{k} = 1 - \frac{{2\mathop \sum \nolimits_{i = 1}^{N} p_{i,k} g_{i,k} }}{{\mathop \sum \nolimits_{i = 1}^{N} p_{i,k} + \mathop \sum \nolimits_{i = 1}^{N} g_{i,k} }}$$

where N, ${\text{g}}_{{{\text{i}},{\text{k}}}}$ and ${\text{p}}_{{{\text{i}},{\text{k}}}}$ are the same as in Formula 1. So we can write the Dice loss for enhance region as:

3 $${\text{D}}L_{enh\_loss} = 1 - \frac{{2\mathop \sum \nolimits_{i = 1}^{N} p_{i,4} g_{i,4} }}{{\mathop \sum \nolimits_{i = 1}^{N} p_{i,4} + \mathop \sum \nolimits_{i = 1}^{N} g_{i,4} }}$$

The Dice loss for background region is as below:

4 $${\text{D}}L_{bg\_loss} = 1 - \frac{{2\mathop \sum \nolimits_{i = 1}^{N} p_{i,0} g_{i,0} }}{{\mathop \sum \nolimits_{i = 1}^{N} p_{i,0} + \mathop \sum \nolimits_{i = 1}^{N} g_{i,0} }}$$

The Dice loss for complete region can be written as:

5 $${\text{D}}L_{com\_loss} = 1 - \frac{{2\mathop \sum \nolimits_{i = 1}^{N} p_{{{\text{i}},{\text{com}}}} g_{{{\text{i}},{\text{com}}}} }}{{\mathop \sum \nolimits_{i = 1}^{N} p_{{{\text{i}},{\text{com}}}} + \mathop \sum \nolimits_{i = 1}^{N} g_{{{\text{i}},{\text{com}}}} }}$$

where ${\text{p}}_{{{\text{i}},{\text{com}}}} = {\text{p}}_{{{\text{i}},4}} + {\text{p}}_{{{\text{i}},3}} + {\text{p}}_{{{\text{i}},1}} + {\text{p}}_{{{\text{i}},2}}$ and ${\text{g}}_{{{\text{i}},{\text{com}}}} = {\text{g}}_{{{\text{i}},4}} + {\text{g}}_{{{\text{i}},3}} + {\text{g}}_{{{\text{i}},1}} + {\text{g}}_{{{\text{i}},2}}$ denote the predicted values and manual segmentation label for complete region, respectively.

The Dice loss for tumor core region can be written as:

6 $${\text{D}}L_{core\_loss} = 1 - \frac{{2\mathop \sum \nolimits_{i = 1}^{N} p_{i,core} g_{i,core} }}{{\mathop \sum \nolimits_{i = 1}^{N} p_{i,core} + \mathop \sum \nolimits_{i = 1}^{N} g_{i,core} }}$$

where ${\text{p}}_{{{\text{i}},{\text{core}}}} = {\text{p}}_{{{\text{i}},4}} + {\text{p}}_{{{\text{i}},3}} + {\text{p}}_{{{\text{i}},1}}$ and ${\text{g}}_{{{\text{i}},{\text{core}}}} = {\text{g}}_{{{\text{i}},4}} + {\text{g}}_{{{\text{i}},3}} + {\text{g}}_{{{\text{i}},1}}$ denote the predicted values and manual segmentation label for tumor core region, respectively.

The combined Dice loss can be defined as:

7 $${\text{D}}L_{combined\_loss} = {\text{D}}L_{bg\_loss} + {\text{D}}L_{com\_loss} + {\text{D}}L_{core\_loss} + {\text{D}}L_{enh\_loss}$$

The sliced Dice loss can be defined as:

8 $${\text{D}}L_{sliced\_loss} = {\text{D}}L_{0} + {\text{D}}L_{1} + {\text{D}}L_{2} + {\text{D}}L_{3} + {\text{D}}L_{4}$$

#### Recall loss

Although model trained with unbalanced data may make high accuracy predictions, the target area may be partially detected or missing, which is very harmful in medical applications. Sensitivity, therefore, can be used to address the imbalance issues, shifting emphasis to the minority.

Sensitivity is one of the highly regarded characteristics when evaluating the performance of image segmentation algorithms. The recall loss for each subclass is as below:

9 $${\text{R}}L_{k} = 1 - \frac{{\mathop \sum \nolimits_{i = 1}^{N} p_{i,k} g_{i,k} }}{{\mathop \sum \nolimits_{i = 1}^{N} g_{i,k} }}$$

where N, ${\text{g}}_{{{\text{i}},{\text{k}}}}$ and ${\text{p}}_{{{\text{i}},{\text{k}}}}$ are the same as in Formula 1. So we can write the recall loss for enhance region as:

10 $${\text{R}}L_{enh\_loss} = 1 - \frac{{\mathop \sum \nolimits_{i = 1}^{N} p_{i,4} g_{i,4} }}{{\mathop \sum \nolimits_{i = 1}^{N} g_{i,4} }}$$

The recall loss for complete region is as below:

11 $${\text{R}}L_{com\_loss} = 1 - \frac{{\mathop \sum \nolimits_{i = 1}^{N} p_{i,com} g_{i,com} }}{{\mathop \sum \nolimits_{i = 1}^{N} g_{i,com} }}$$

where ${\text{p}}_{{{\text{i}},{\text{com}}}} = {\text{p}}_{{{\text{i}},4}} + {\text{p}}_{{{\text{i}},3}} + {\text{p}}_{{{\text{i}},1}} + {\text{p}}_{{{\text{i}},2}}$ and ${\text{g}}_{{{\text{i}},{\text{com}}}} = {\text{g}}_{{{\text{i}},4}} + {\text{g}}_{{{\text{i}},3}} + {\text{g}}_{{{\text{i}},1}} + {\text{g}}_{{{\text{i}},2}}$ denote the predicted values and manual segmentation label for complete region, respectively.

The recall loss for tumor core region is as below:

12 $${\text{R}}L_{core\_loss} = 1 - \frac{{2\mathop \sum \nolimits_{i = 1}^{N} p_{i,core} g_{i,core} }}{{\mathop \sum \nolimits_{i = 1}^{N} g_{i,core} }}$$

where ${\text{p}}_{{{\text{i}},{\text{core}}}} = {\text{p}}_{{{\text{i}},4}} + {\text{p}}_{{{\text{i}},3}} + {\text{p}}_{{{\text{i}},1}}$ and ${\text{g}}_{{{\text{i}},{\text{core}}}} = {\text{g}}_{{{\text{i}},4}} + {\text{g}}_{{{\text{i}},3}} + {\text{g}}_{{{\text{i}},1}}$ denote the predicted values and manual segmentation label for tumor core categories, respectively.

#### Hybrid loss

We design two hybrid loss functions, which include recall loss, combined Dice loss and cross-entropy loss, to better balance recall and precision. The first one named HL1 can be written as:

13 $${\text{H}}L_{ce\_\_rl\_loss} = \alpha L_{ce\_loss} + \beta {\text{R}}L_{com\_loss} + \gamma {\text{R}}L_{core\_loss} + \delta {\text{R}}L_{enh\_loss}$$

where ${\updelta },$ ${\upalpha },$ ${\upbeta }$ and ${\upgamma }$ are weights of different loss.

The second one named HL2 can be written:

14 $${\text{H}}L_{cb\_\_rl\_loss} = \alpha {\text{D}}L_{combined\_loss} + \beta {\text{R}}L_{com\_loss} + \gamma {\text{R}}L_{core\_loss} + \delta {\text{R}}L_{enh\_loss}$$

where ${\updelta },$ ${\upalpha },$ ${\upbeta }$ and ${\upgamma }$ are weights of different loss.

## Results

### Evaluation metrics

In this study, the online system provided by the Brats challenge is used to evaluate our results. It evaluates the results from sensitivity, PPV (positive predictive value) and DSC for the three tumor regions (complete, core, and enhancing).

DSC is applied to measure the intersection between the regions predicted by the model and the regions segmented by the human. A higher DSC value means a better performance. The DSC is defined:

15 $$DSC\left( {P,G} \right) = \frac{{2\left| {P \cap G} \right|}}{\left| P \right| + \left| G \right|}$$

where P denotes the predicted segmentation, and G is the manual segmentation.

PPV is applied to measure the overlap percentage between the regions predicted by the model and the regions segmented by the human to the predicted regions, and can be defined with the following Eq. (16):

16 $$PPV\left( {P,G} \right) = \frac{{\left| {P \cap G} \right|}}{\left| P \right|}$$

Sensitivity is applied to measure the overlap percentage between the regions predicted by the model and the regions segmented by the human to the manual segmentation regions. It is defined as Eq. (17):

17 $$Sensitivity\left( {P,G} \right) = \frac{{\left| {P \cap G} \right|}}{\left| G \right|}.$$

### Implementation details

We build our network in Keras (TensorFlow as backend). Our model is trained with Adam optimizer on two NVIDIA GPUs (GTX 1080Ti). The batch size is 40. During the training, we use Cyclical learning rates [36] to adjust the learning rate. In each cycle, the learning rates are set to 0.000001, 0.001 for minimum and maximum learning rates. For data augmentation, we adopt flip and rotation from the training set to ease the over-fitting issue.

### Evaluating the effectiveness of the proposed model

We adopt U-net as our baseline. For fairly comparing and avoid the improvement is because of more parameters of our proposed model, we change the number of filter channels to be 32, 64, 128, 256 and 512 for each stage in U-net. The capacity of our proposed model (the size of parameters: 6.3 MB) is smaller than that of U-net (the size of parameters: 30.3 MB). Also, we note that the neural network trained on different loss functions exhibits different segmentation performance, so we design three groups of experiments to test our proposed model.

As shown in Table 1, each group is optimized with different loss functions. For group one (first two rows), the cross-entropy loss is used to optimize both U-net and the proposed model. The Dice score of U-net for the three tumor regions (complete, core and enhancing) are 0.84, 0.66 and 0.58. Our proposed model achieves with Dice score of 0.85, 0.67, and 0.59. It is obvious that the proposed model is superior to U-net in all tumor categories. For the second group (two rows in the middle), sliced Dice loss is used to guide the optimization process. Our model outperforms U-net in two categories (core and complete) and gets comparable performance in enhancing category. In the last group (the last two rows), both U-net and our proposed model are optimized with combined Dice loss. Our proposed model yields better DSC scores (0.85, 0.69, 0.59) for all tumor categories. The proposed model also gets higher sensitivity scores and competitive PPV results in all tumor regions. From the above results, our proposed method has high efficiency in the experiments and boosts the performance for its effective feature extraction and feature fusion strategy.

**Table 1:** Performance comparison between our proposed model and U-net

Method	Loss function	DSC			PPV			Sensitivity		
		Complete	Core	Enh	Complete	Core	Enh	Complete	Core	Enh
U-net	Cross entropy loss	0.84	0.66	0.58	0.87	0.80	0.60	0.84	0.61	0.61
proposed		0.85	0.67	0.59	0.87	0.83	0.60	0.85	0.62	0.64
U-net	Sliced Dice loss	0.83	0.64	0.56	0.87	0.82	0.63	0.82	0.60	0.57
Proposed		0.84	0.66	0.56	0.86	0.83	0.56	0.85	0.62	0.54
U-net	Combined Dice loss	0.83	0.65	0.55	0.87	0.83	0.65	0.83	0.59	0.54
proposed		0.85	0.69	0.59	0.86	0.82	0.64	0.87	0.65	0.60

Each group was optimized with different loss functions

**Fig. 5:**
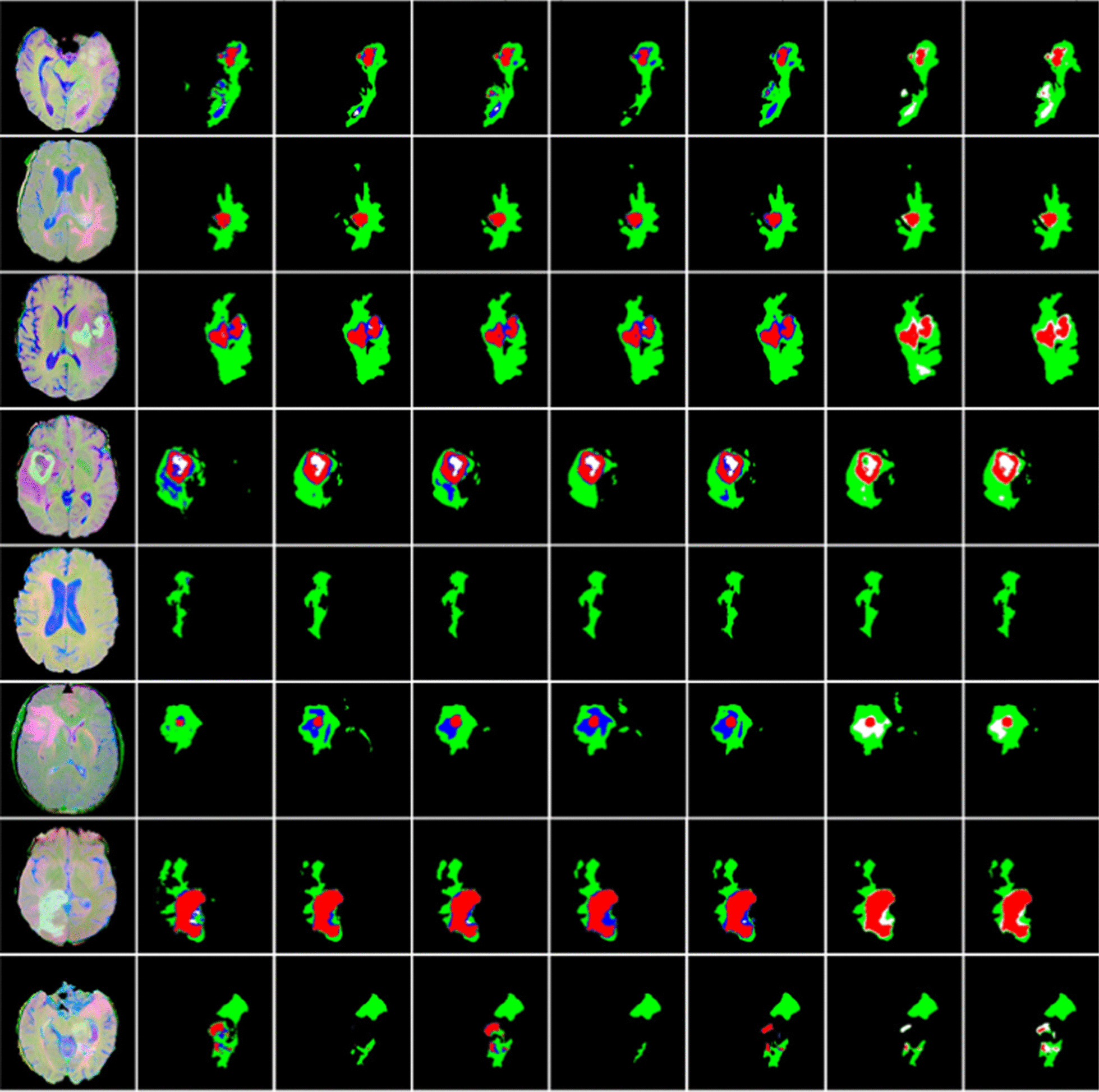
Visualized comparison between our proposed method and U-net on BRATS 2015 validation set (best view in color). There are three groups of experiments to verify the effectiveness of our proposed model. The first column shows images generated by T1c, T2 and FLAIR with a merge operation. The second column shows the ground truth. The first group (the third and fourth column) shows the segmentation results generated by U-net and the proposed method with cross-entropy loss function. The second group (the fifth and sixth column) shows the segmentation results generated by U-net and the proposed method with sliced Dice loss function. The last group (the last two columns) shows the segmentation results generated by U-net and the proposed method with combined Dice loss function. Pixels labeled in black are background in the last five columns. Each of the other colors represents a tumor region: necrosis (blue), edema (green), non-enhancing (white) and enhancing (red)

Additionally, the corresponding qualitative comparison is shown in Fig. 5. It is obvious that the segmentation results generated by our proposed model are more consistent with the manual annotation in most cases. With ground truth as a reference, our proposed method generates more accurate results than U-net, especially for the border of the tumor. For the last group (the last two columns), we note that both U-net and our proposed model cannot distinguish between necrosis region (blue) and non-enhancing region (white) because of the combined Dice loss function, which is designed for the three different tumor regions required by the online evaluation system. Moreover, it is noted that the output behavior of the segmentation network is changed by different loss functions.

### Evaluating the effectiveness of hybrid loss function

The proposed model is used to verify the segmentation effect of two hybrid loss functions. For the convenience of comparison, we divide those experiments into two groups. First, we compare the cross-entropy loss with the first hybrid loss (HL1). Second, we compare the combined Dice loss with the second hybrid loss (HL2).

For the first hybrid loss function (HL1), we set ${\upalpha }=20,$ $\upbeta =0.5 ,\upgamma =1$ and $\updelta =0.5$ as they provided the best results. As seen in Table 2 (the first two rows), we note that the model trained with first hybrid loss (HL1) performs better than the one trained with cross-entropy for all tumor categories. For tumor core category, the first hybrid loss significantly increases the Dice score by 4%. The recall loss in the first hybrid loss helps the network to achieve a better result, which can be seen from the comparison that sensitivity significantly increase at the expense of a decrease in precision, while maintaining the Dice score unchanged or increased.

**Table 2:** Performance comparison of our proposed method with different loss function on the test set

Loss function	DSC			PPV			Sensitivity		
	Complete	Core	Enh	Complete	Core	Enh	Complete	Core	Enh
cross entropy	0.85	0.67	0.59	0.87	0.83	0.60	0.85	0.62	0.64
$\mathrm{H}{L}_{ce\_\_rl\_loss}$	0.85	0.71	0.60	0.83	0.76	0.57	0.89	0.73	0.68
combined Dice loss	0.85	0.69	0.59	0.86	0.82	0.64	0.87	0.65	0.60
$\mathrm{H}{L}_{cb\_\_rl\_loss}$	0.85	0.71	0.59	0.85	0.75	0.58	0.87	0.74	0.65

We also test the performance of the second hybrid loss (HL2) against the combined Dice loss function (the last two rows). For the second hybrid loss function (HL2), we set ${\upalpha }=1,$ $\upbeta =0.1 ,\upgamma =1.3$ and $\updelta =0.5$ as they provided the best results. The model trained with HL2 outperforms the one trained with combined Dice loss in the tumor core and enhancing region and achieves comparable performance on complete region. For tumor core category, the second hybrid loss also increases the Dice score by 2%. Similarly, sensitivity and PPV in the second group are consistent with the first group, with a significant increase in sensitivity at the expense of a decrease in PPV. Particularly, our proposed method trained with hybrid loss function obtain better sensitivity in all tumor regions, showing that the hybrid loss function can identify tumor areas from non-tumor areas. The above results reveal the effectiveness of the hybrid loss function and the potential to balance recall and precision.

### Comparison to other published best methods

Our approach is not suitable for direct comparison with these methods proposed in the BRATS 2015. During that challenge, only 53 cases are used for testing, but now the online evaluation system provides 110 testing cases. Our proposed method is compared with two published best approaches. The first one is a patch-based method [11]. They train three different models for segmentation, each using patches selected from different views of MRI volume. They then apply the models to do the segmentation in axial, coronal and sagittal directions. Finally, the final result is fused from the three different segmentation results with a majority vote strategy. The second one is also a patch-based approach [9], including a 3D CNN (DeepMedic) and 3D CRF. This approach simultaneously processes multiple scale input images with two pathway architecture and can combine local and broader context information. Their ensemble is built with three base models, applying an average strategy to aggregate different segmentation results.

To allow a fair comparison, all segmentation results listed are obtained by a single model. As seen in Table 3, we have the following observations. First, all methods achieve a low segmentation performance in enhancing tumor region. This mainly contributes to the difficulties incurred by the small size of the target region in the brain. Second, our proposed method outperforms both DeepMedic and DeepMedic with 3D CRF in compete tumor and tumor core region. Third, compared with FCNN with CRF proposed by Zhao et al., our method exhibits superior performance for all tumor categories. Fourth, our method is comparable or superior to FCNN with 3D CRF in compete tumor and tumor core region, except the enhancing tumor region. From the above comparison, we find that FCNN with 3D CRF significantly improves the segmentation performance in comparison of FCNN with CRF in all tumor regions, demonstrating 3D CRF is the key to the performance increase. We can conclude that the feature representations of our proposed method are more powerful than FCNN designed by Zhao et al. Compared with other published works, our performance is equal to or better than that of other methods, without any post-processing operations. In most cases, the proposed method has high sensitivity, showing that it can effectively identify tumor areas from non-tumor areas.

**Table 3:** Performance comparison of different single models on the test set

Method	DSC			PPV			Sensitivity		
	Complete	Core	Enh	Complete	Core	Enh	Complete	Core	Enh
DeepMedic [12]	0.836	0.674	0.629	0.823	0.846	0.64	0.885	0.616	0.656
DeepMedic + CRF [12]	0.847	0.67	0.629	0.85	0.848	0.634	0.876	0.607	0.662
FCNN + CRF (axial) [13]	0.78	0.64	0.54	0.78	0.76	0.48	0.81	0.62	0.71
FCNN + CRF (coronal) [13]	0.77	0.66	0.56	0.73	0.73	0.52	0.86	0.67	0.67
FCNN + CRF (sagittal) [13]	0.76	0.63	0.47	0.75	0.71	0.38	0.80	0.63	0.75
FCNN + 3D CRF (axial) [13]	0.84	0.72	0.62	0.88	0.75	0.62	0.82	0.76	0.67
FCNN + 3D CRF(coronal) [13]	0.84	0.72	0.62	0.88	0.75	0.62	0.82	0.75	0.66
FCNN + 3D CRF (sagittal) [13]	0.82	0.72	0.60	0.88	0.75	0.59	0.81	0.76	0.67
Proposed + $\mathrm{H}{L}_{cb\_\_rl\_loss}$	0.85	0.71	0.59	0.85	0.75	0.58	0.87	0.74	0.65
Proposed + $\mathrm{H}{L}_{ce\_\_rl\_loss}$	0.85	0.71	0.60	0.83	0.76	0.57	0.89	0.73	0.68

We develop an ensemble comprising five models based on our proposed architectures trained with hybrid loss. Specifically, the BRATS 2015 dataset is split into two parts. There are 274 cases available. 20% of the data is for validation and 80% for training in each split. We train each model with different training sets and select the best weights on the validation loss. We can see from Tables 3 and 4 that our ensemble model outperforms the single model in Dice score and achieves competitive results in the other two evaluation metrics (PPV, sensitivity). This implies that ensemble learning that aggregates different models improves segmentation performance. As shown in Table 4, we compare our ensemble to other ensemble methods. The result of our ensemble method is better than other ensemble methods on the complete tumor region, and it is comparable on other categories. Remarkably, our ensemble method obtains the best Dice similarity coefficient of 0.86 in the complete tumor region.

**Table 4:** Performance comparison of different ensemble models on the test set

Method	DSC			PPV			Sensitivity		
	Complete	Core	Enh	Complete	Core	Enh	Complete	Core	Enh
DeepMedic (ensemble) [12]	0.845	0.667	0.633	0.833	0.861	0.632	0.889	0.599	0.673
DeepMedic + CRF (ensemble) [12]	0.849	0.667	0.634	0.853	0.861	0.634	0.877	0.600	0.674
FCNN + 3D CRF (fusing) [13]	0.84	0.73	0.62	0.89	0.76	0.63	0.82	0.76	0.67
Proposed + $\mathrm{H}{L}_{cb\_\_rl\_loss}$	0.86	0.73	0.61	0.86	0.76	0.60	0.88	0.76	0.65
Proposed + $\mathrm{H}{L}_{ce\_\_rl\_loss}$	0.86	0.72	0.60	0.86	0.76	0.56	0.88	0.74	0.70

## Discussion

Although remarkable improvements have made in past years, the deep learning based methods still have some challenges, such as good feature representations and effective training processes on the severe class imbalance dataset, which limit the performance and generalization ability. It is promising to design more powerful multi-scale feature extraction and feature fusion techniques and construct an effective way to guide the training process on an imbalanced dataset.

The first challenging is that MRI images usually have a poor image quality, such as a low contrast between different tissue regions and tumor regions with high boundary-uncertainty. Moreover, tumors have irregular shapes from case to case, which makes it hard to segment the different tumor regions. To the end, we propose a multi-scale feature extraction and feature fusing mechanism for rich representative features. Although the capacity of our proposed model is smaller than that of the U-net, we still achieve better segmentation results. We attribute the performance boost to the advantage of the multi-scale feature extraction and feature fusing mechanism, showing its ability to capture more representative features.

Capturing more representative features is the key to more accurate segmentation. Although 2D CNN with different directions (axial, coronal, and sagittal) or 3D CNN could be beneficial for a rich feature representation, which increases the computational costs and memory loads. The information captured by each convolutional layer in the feature extraction network is useful in feature representation and should use as much as possible. For example, shallow feature maps from early convolutional layers have detail information but poor semantic information, which may benefit object segmentation performance, especially for small objects. This is the reason we take all the feature maps from early convolutional layers. The results in Fig. 5 also suggest that features from the early convolutional layers are more beneficial for preserving fine details of the tumor regions.

The core idea of our feature fusion mechanism is that there is a complementary relationship between different-level features. To fuse features more effectively, we concatenate features maps and fuse the different-level features in a parallel way, which is expected to take full advantage of these features and generate rich feature representation in both detail and semantic information. Compared with U-net, the proposed model achieves better performance for the three tumor regions. We believe such a mechanism benefits the feature fusing processes and is the main reason for the performance gain. Moreover, the multi-scale feature maps are up-sampled and fed into the fusing block, so when training the network, the gradient is easier to spread to the deeper convolution layer, which is conducive to the learning of the whole network. Hence, more multi-scale features can be extracted from the feature extraction network. On the other hand, the direct up sampling feature maps will cause more GPU memory cost, so the GPU inference speed of our model is a little slower than of u-net, but its impact on performance is limited and does not affect the application.

Another challenge is class imbalance. We confront such optimization issues during the training, so finding an effective way to guide the training process is needed. Seen from Table 2, the two hybrid loss functions alleviate the class imbalance issue, especially for the core tumor region. In particular, the proposed method trained with hybrid loss function achieves superior sensitivity values in all tumor regions, suggesting that the hybrid loss function can identify tumor areas from non-tumor areas and reveal the potential to control balance precision and recall.

Although the compared methods achieve better performance in enhancing tumor region, the improvement is because of using 3D patches or using 3D CRF. Compared with 2D FCNN model proposed by Zhao et al., our method exhibits superior performance (0.59 vs. 0.54) in enhancing tumor region, suggesting its ability to capture more representative features than FCNNs. This implies that there is potential room for combining 3D CRF to improve the performance for future studies.

## Conclusions

We design a simple parallel CNN to segment brain tumor of MRI images. The parallel structure in the network can effectively fuse multi-level features to form rich feature representation generate high-resolution results. For the class imbalance issue, we design two hybrid loss functions to guide the training process, which can control the balance precision and recall. Experimental results achieve promising performance without any post-processing operations, showing the effectiveness of our model and the hybrid loss functions. It would be interesting to explore our proposed method in other medical segmentation tasks.

## Data Availability

The datasets analyzed during the current study are available in the SICAS Medical Image Repository, https://www.smir.ch/BRATS/Start2015.

## Abbreviations

BRATS: Multimodal brain tumor image segmentation benchmark
CNN: Convolutional neural network
MRI: Magnetic resonance imaging
FCNN: Fully convolutional networks
CRF: Conditional random fields
DSC: Dice similarity coefficient
PPV: Positive predictive value
FEN: Feature extraction network
MSFFN: Multi-scale feature fusing network
FCN: Fully convolutional networks
ASPP: Atrous spatial pyramid pooling
ReLU: Rectified linear unit
BN: Batch normalization
